# Supplementary figures and images for: Achieving Occam’s razor: Deep learning for optimal model reduction
Source: PLoS Comput Biol. 2024 Jul 18;20(7):e1012283. doi: 10.1371/journal.pcbi.1012283 (PMC11288447; doi:10.1371/journal.pcbi.1012283)

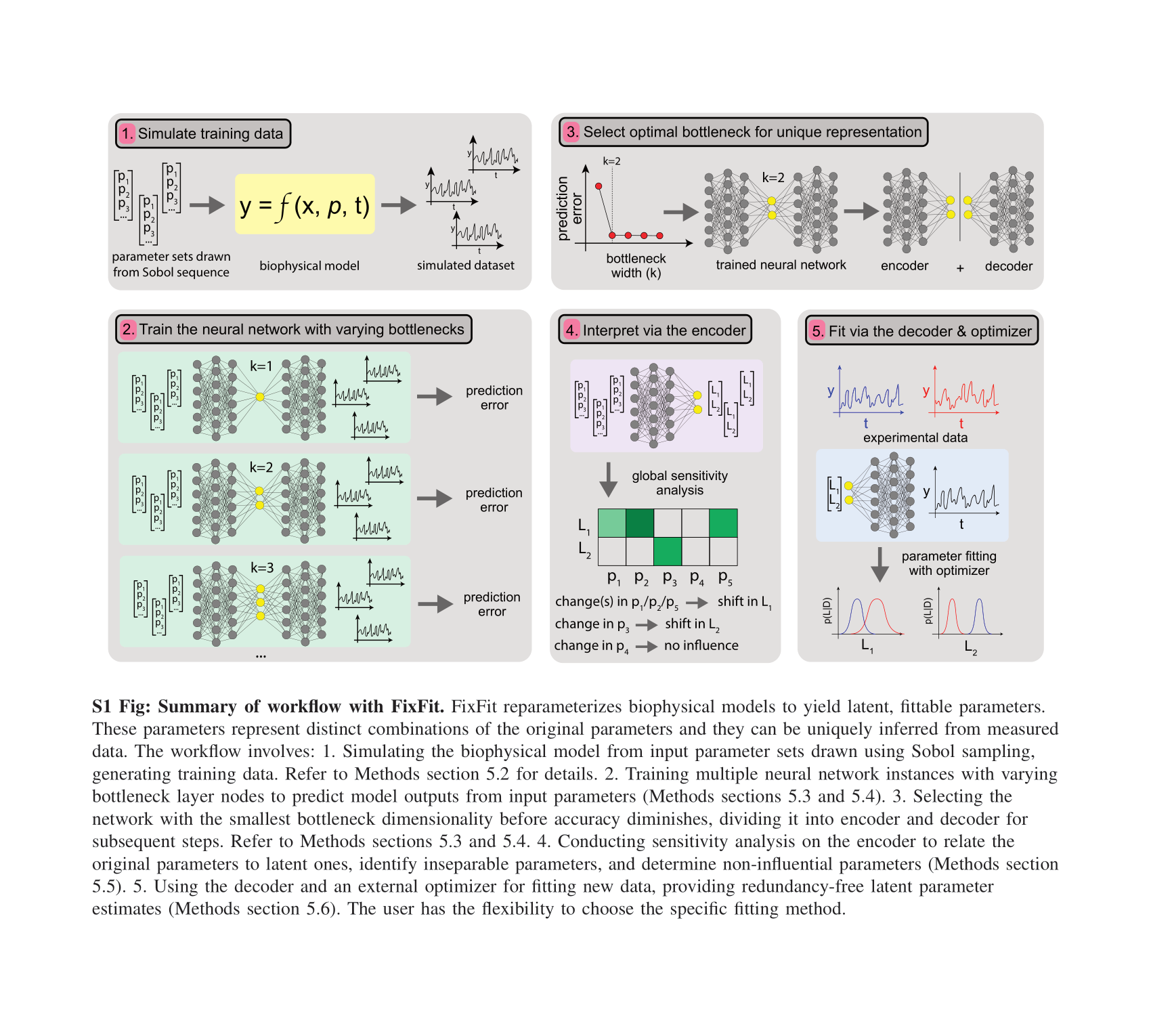

Supplement: S1 Fig — (TIFF) [file pcbi.1012283.s001.tiff]

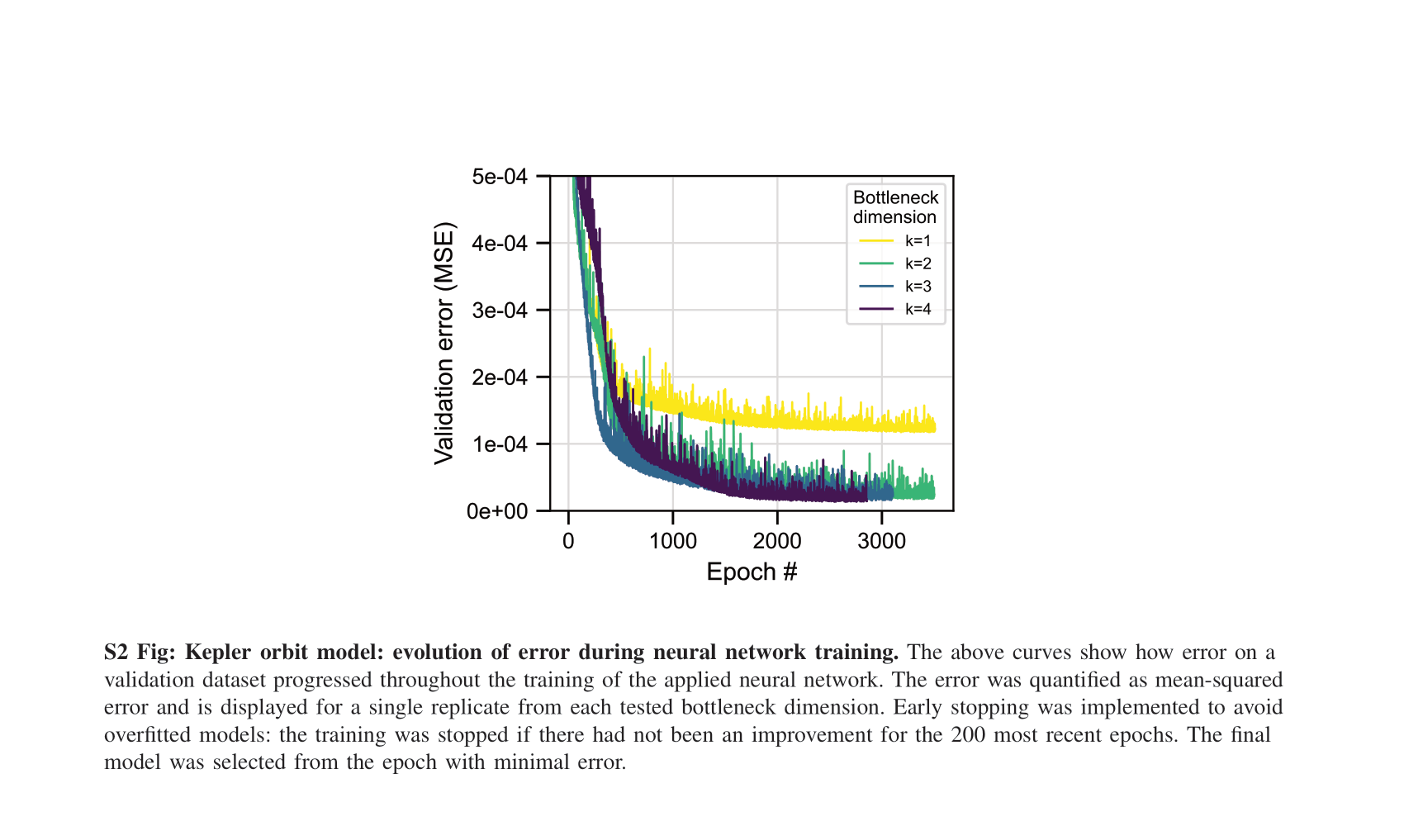

Supplement: S2 Fig — (TIFF) [file pcbi.1012283.s002.tiff]

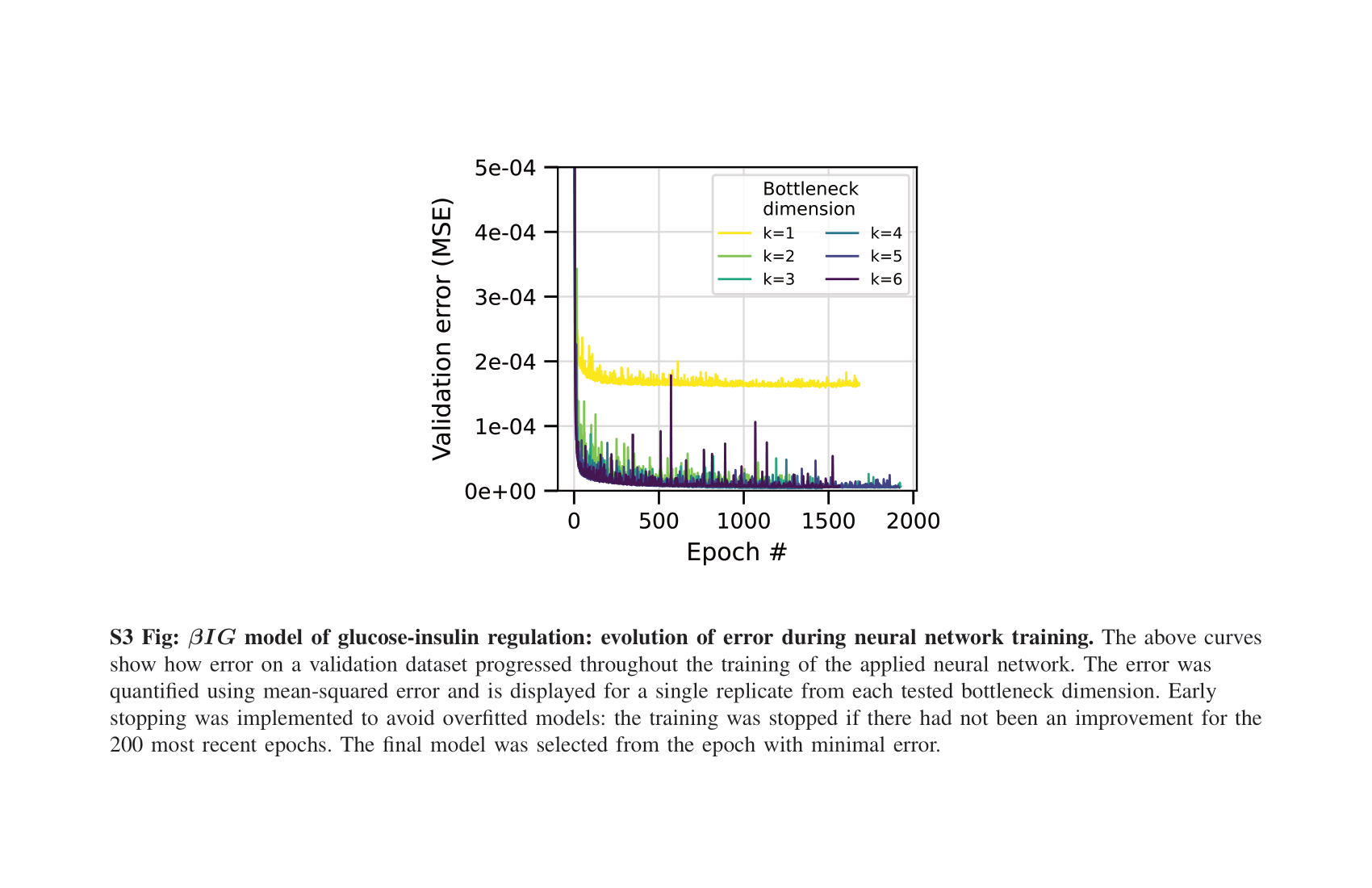

Supplement: S3 Fig — (TIFF) [file pcbi.1012283.s003.tiff]

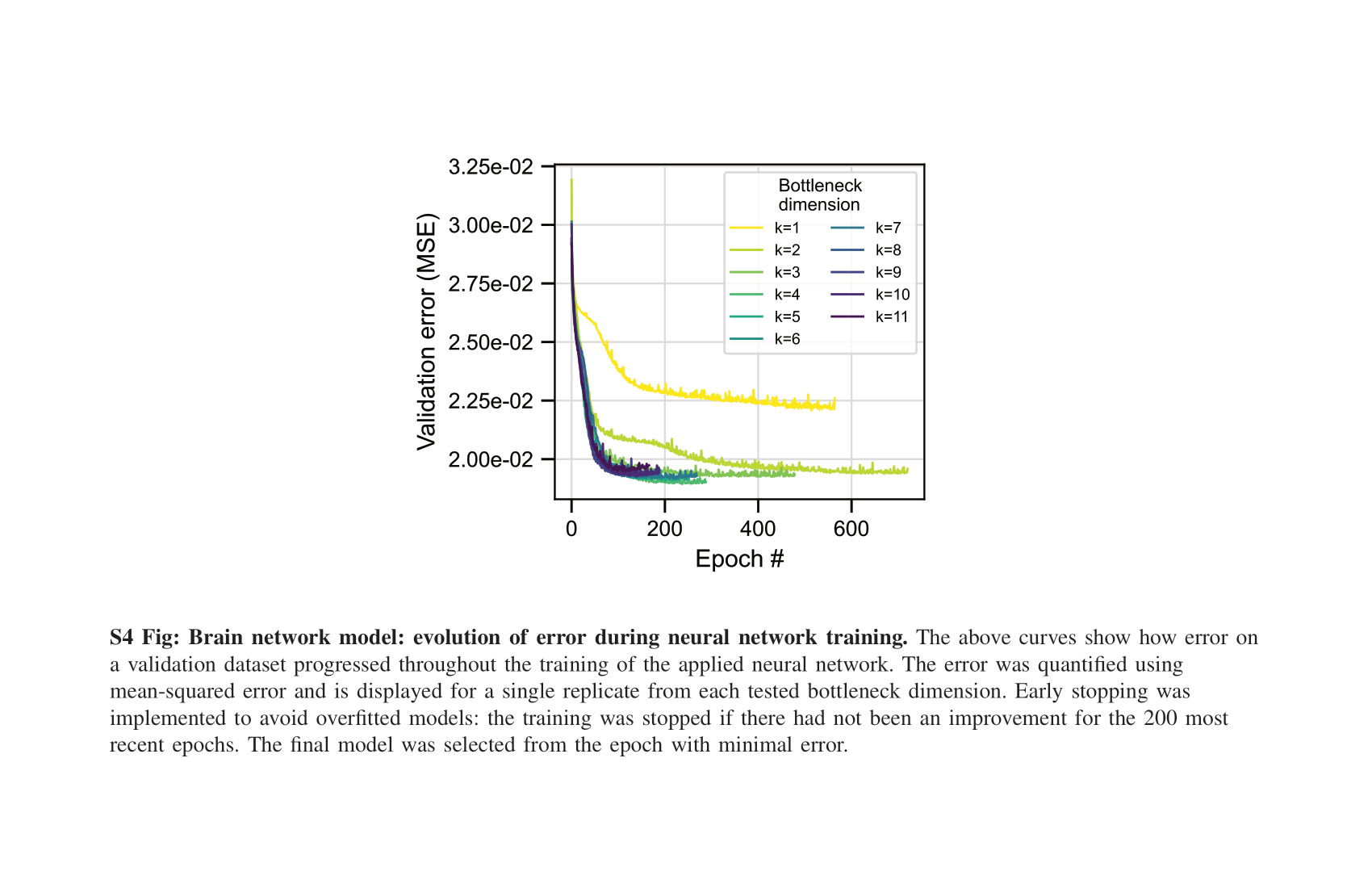

Supplement: S4 Fig — (TIFF) [file pcbi.1012283.s004.tiff]

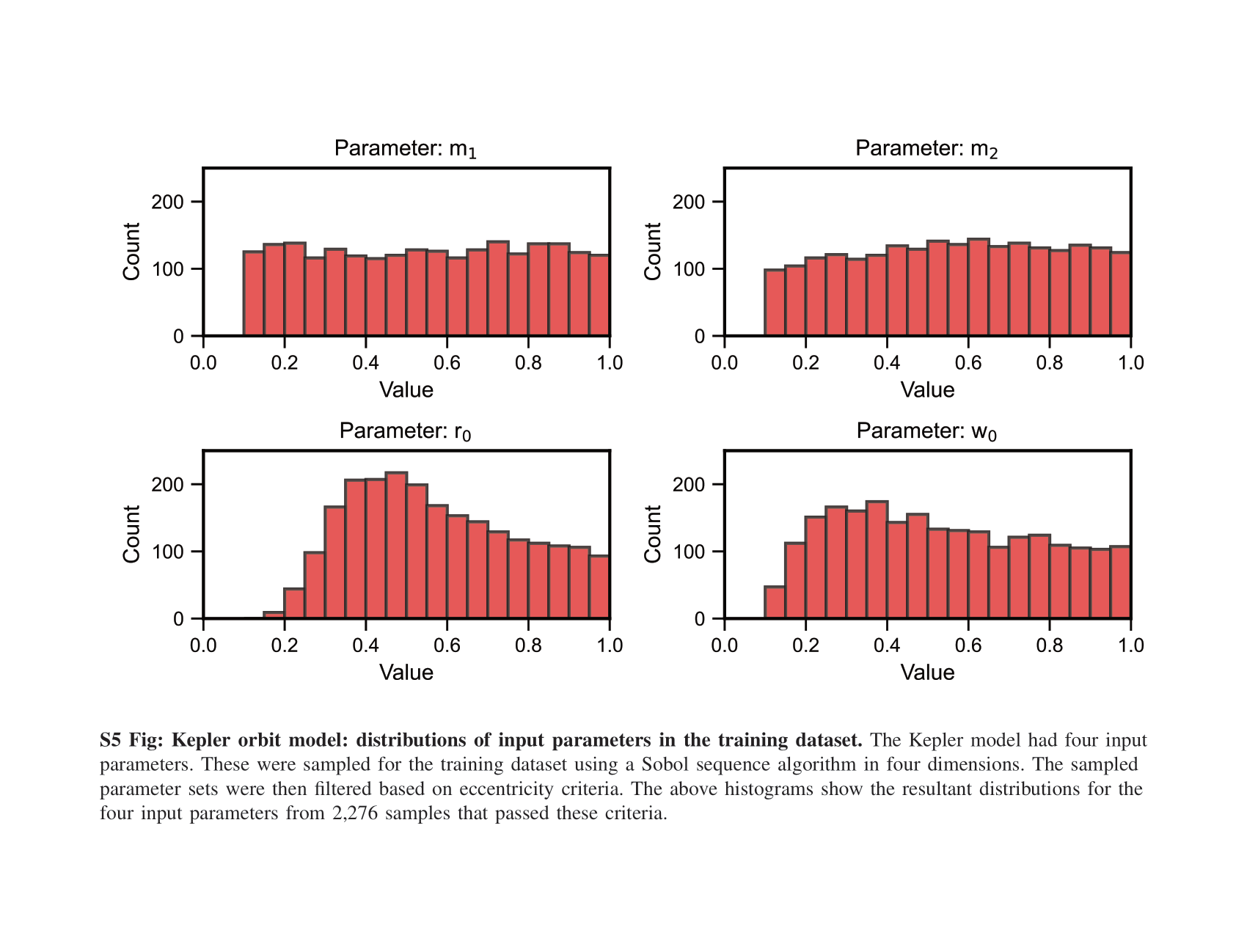

Supplement: S5 Fig — (TIFF) [file pcbi.1012283.s005.tiff]

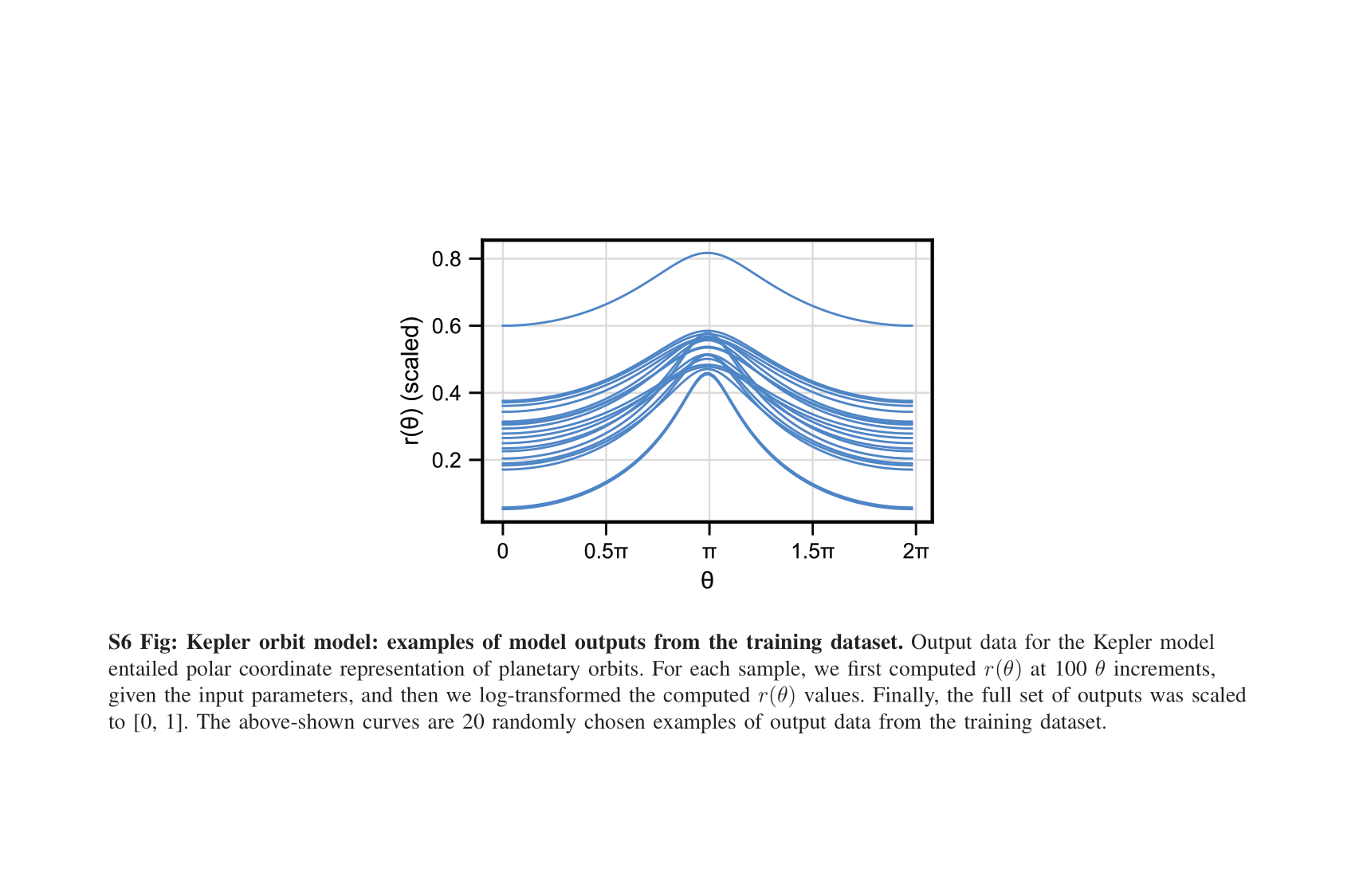

Supplement: S6 Fig — (TIFF) [file pcbi.1012283.s006.tiff]

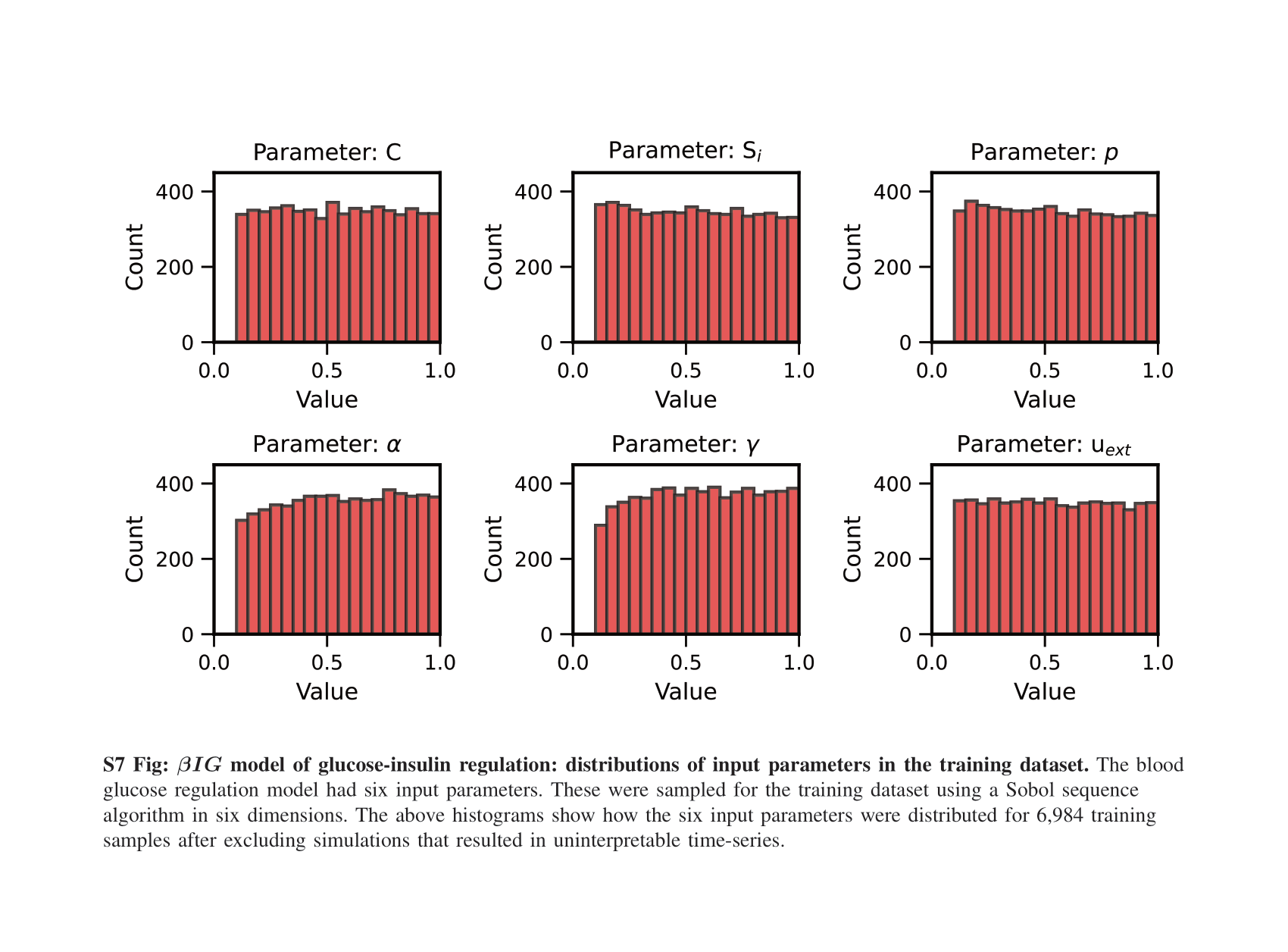

Supplement: S7 Fig — (TIFF) [file pcbi.1012283.s007.tiff]

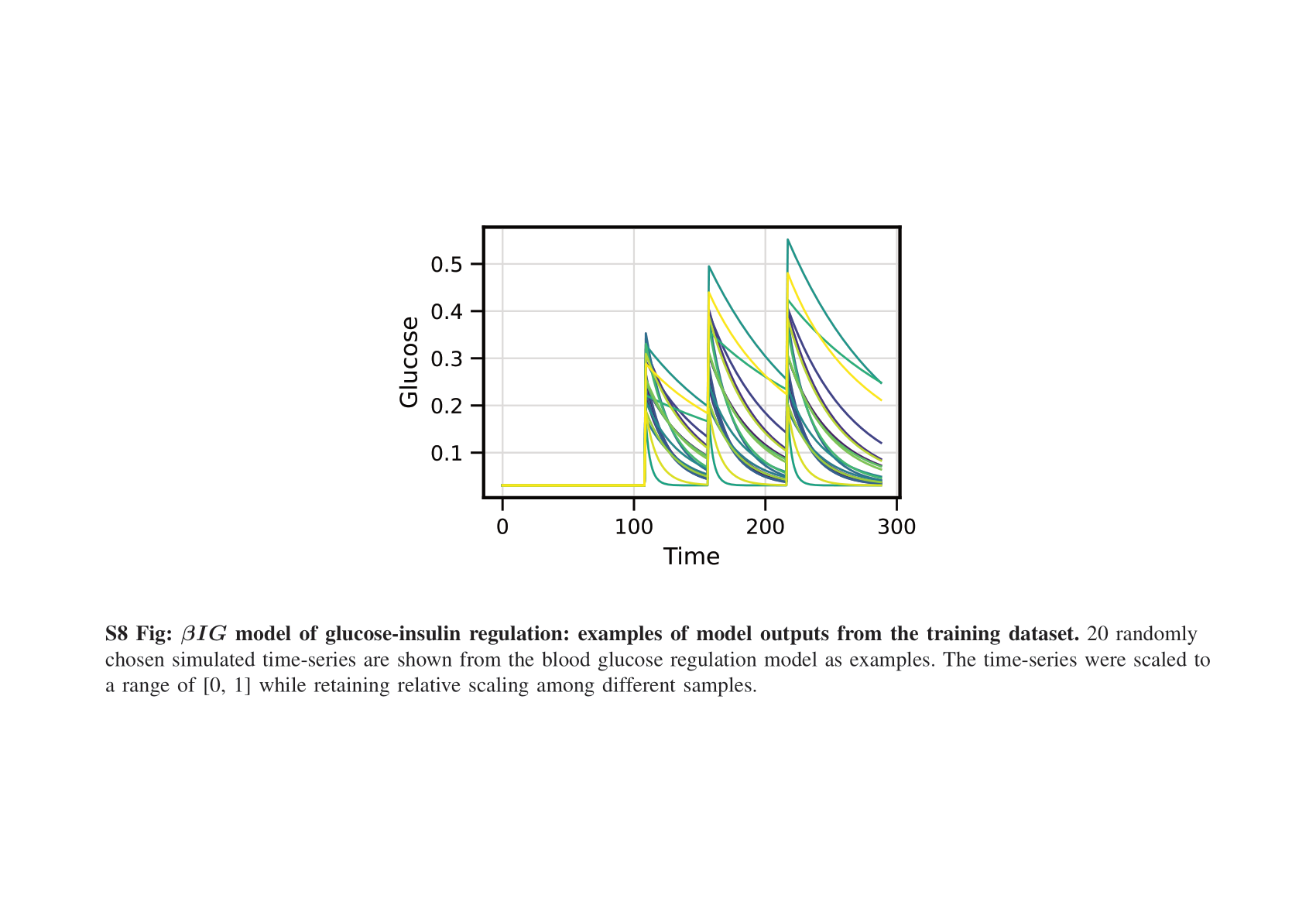

Supplement: S8 Fig — (TIFF) [file pcbi.1012283.s008.tiff]

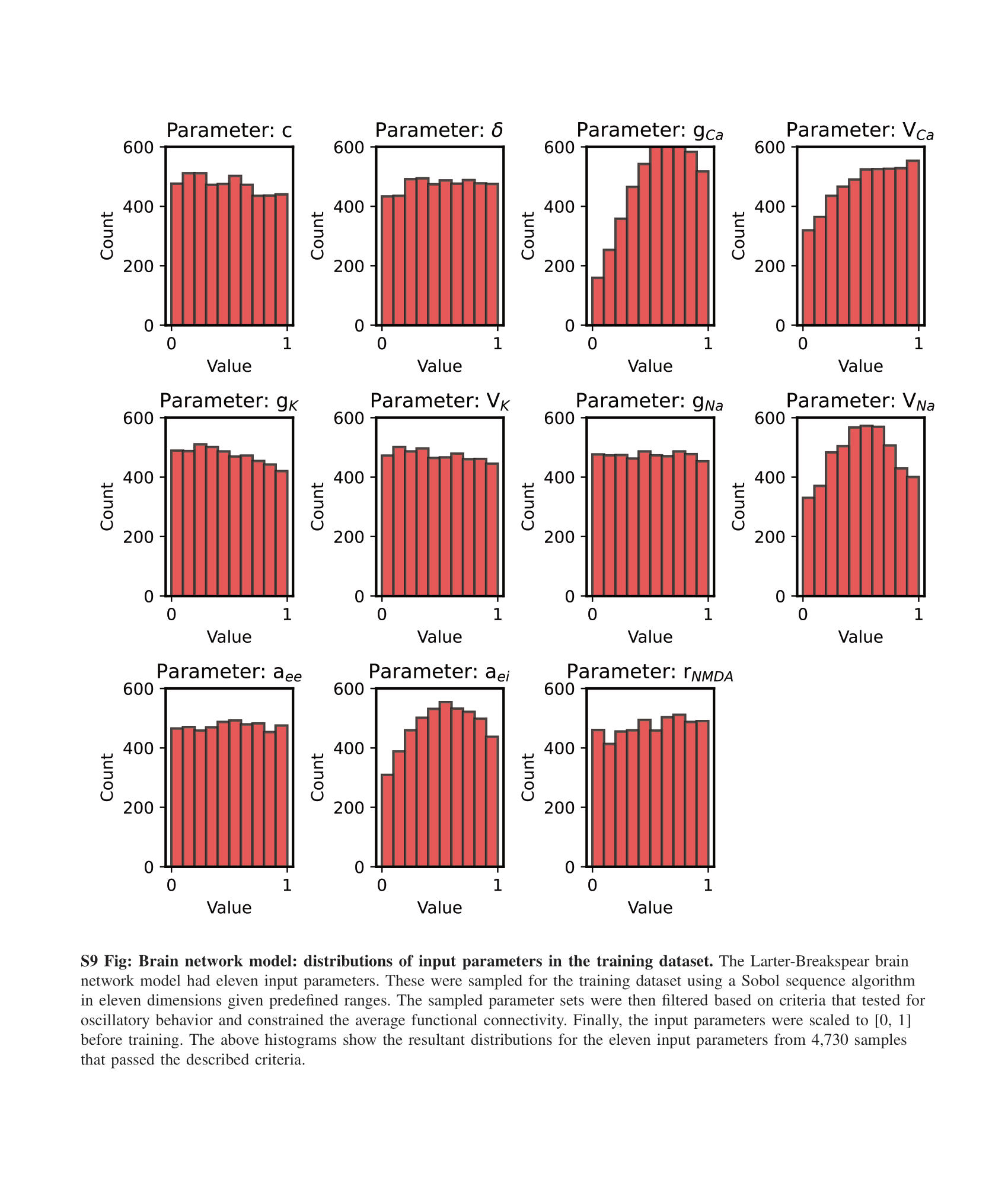

Supplement: S9 Fig — (TIFF) [file pcbi.1012283.s009.tiff]

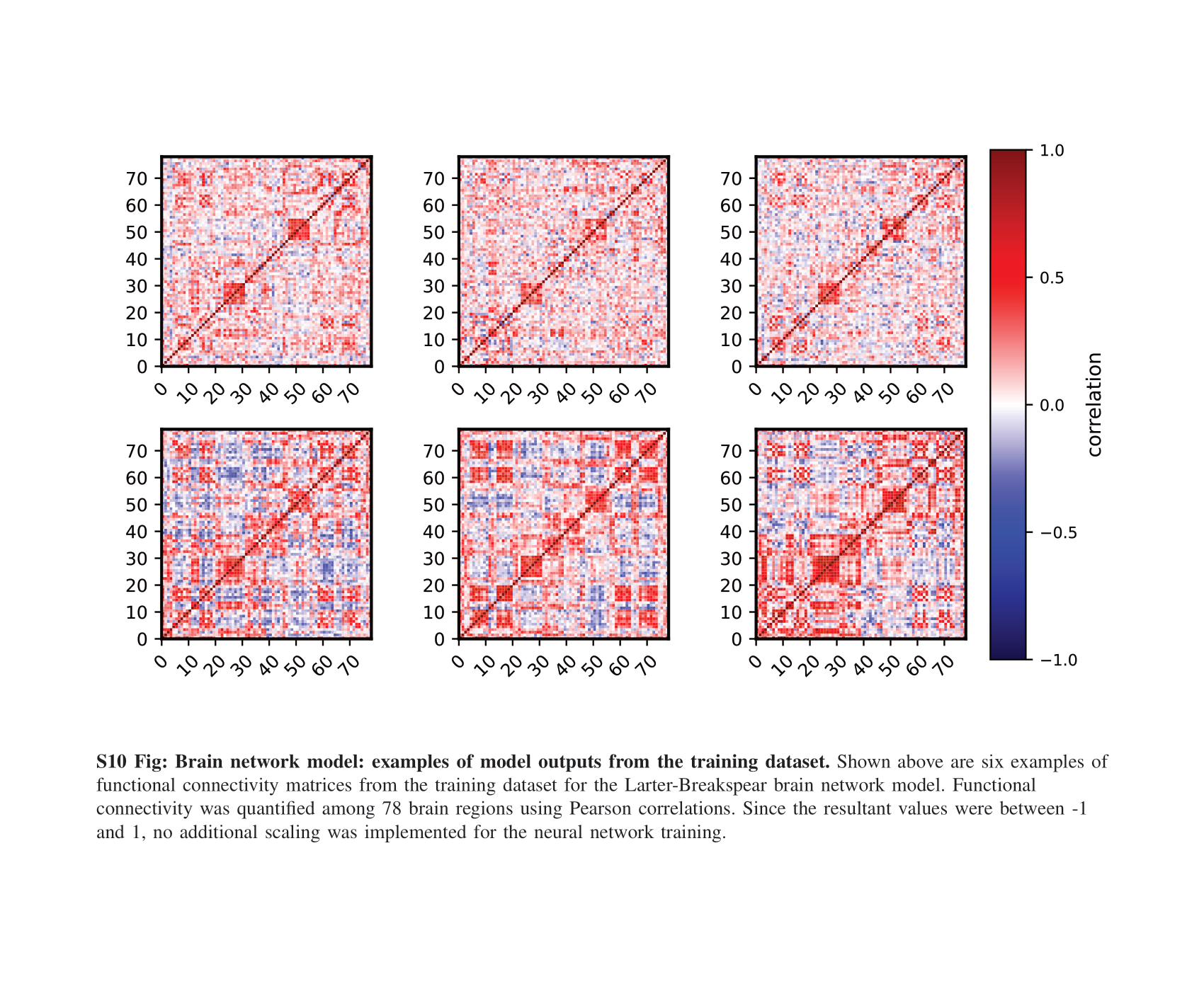

Supplement: S10 Fig — (TIFF) [file pcbi.1012283.s010.tiff]

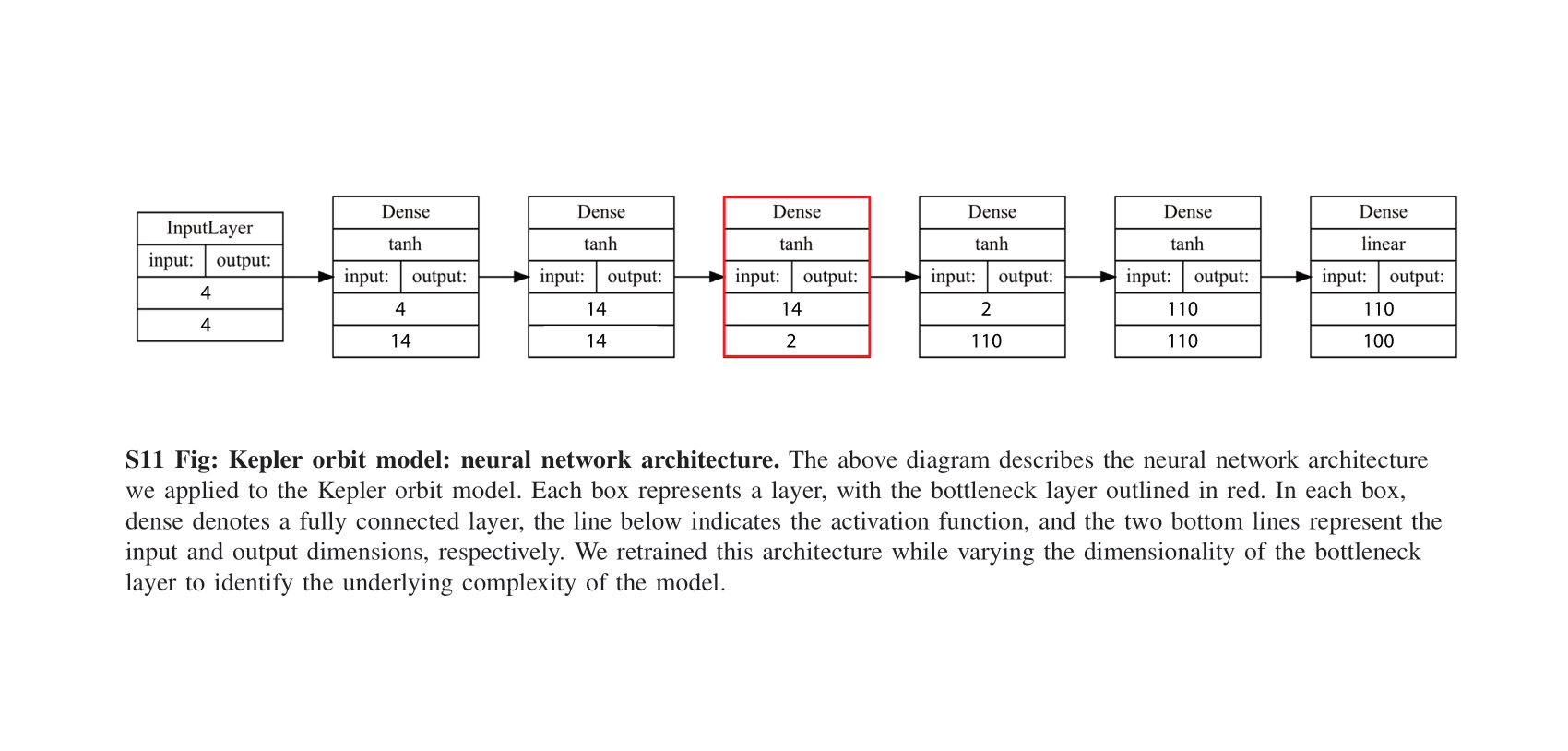

Supplement: S11 Fig — (TIFF) [file pcbi.1012283.s011.tiff]

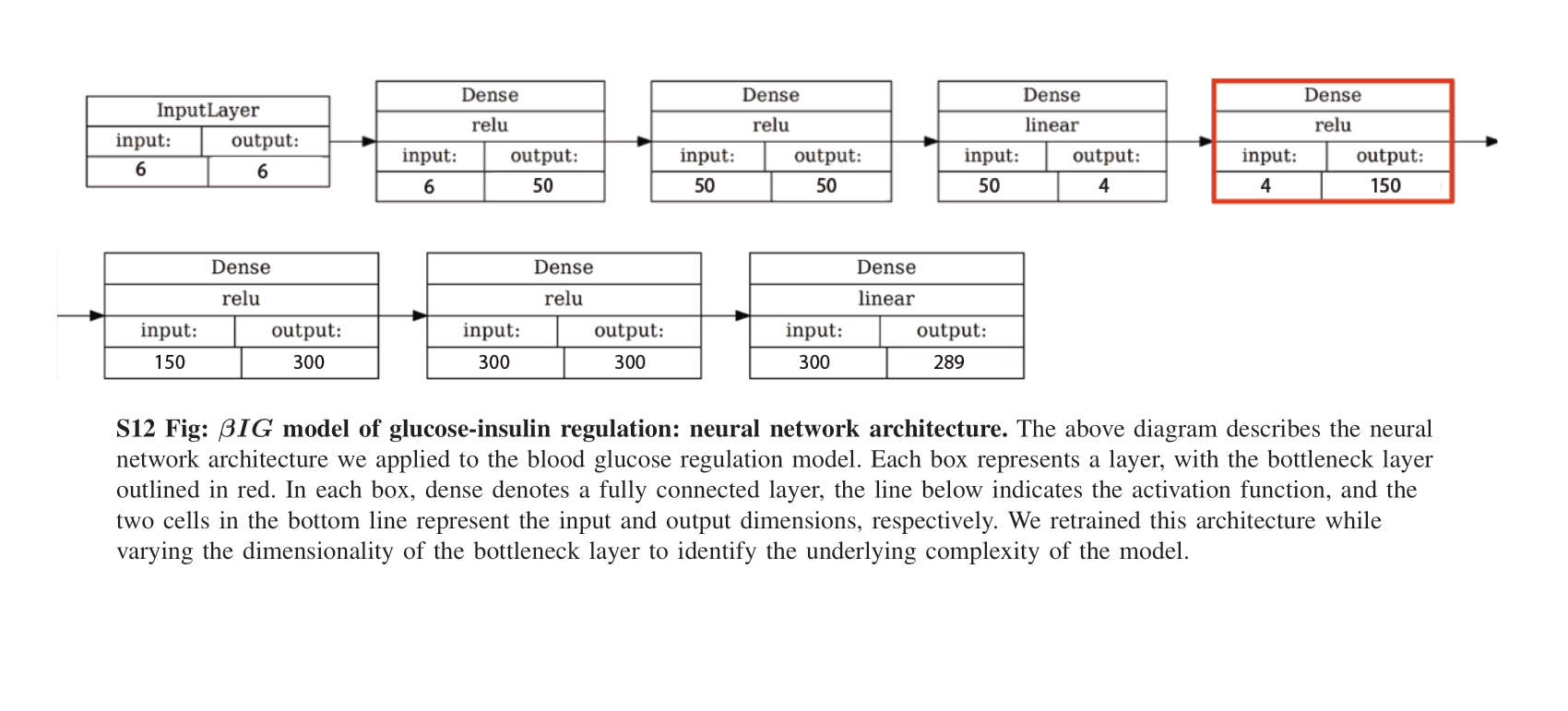

Supplement: S12 Fig — (TIFF) [file pcbi.1012283.s012.tiff]

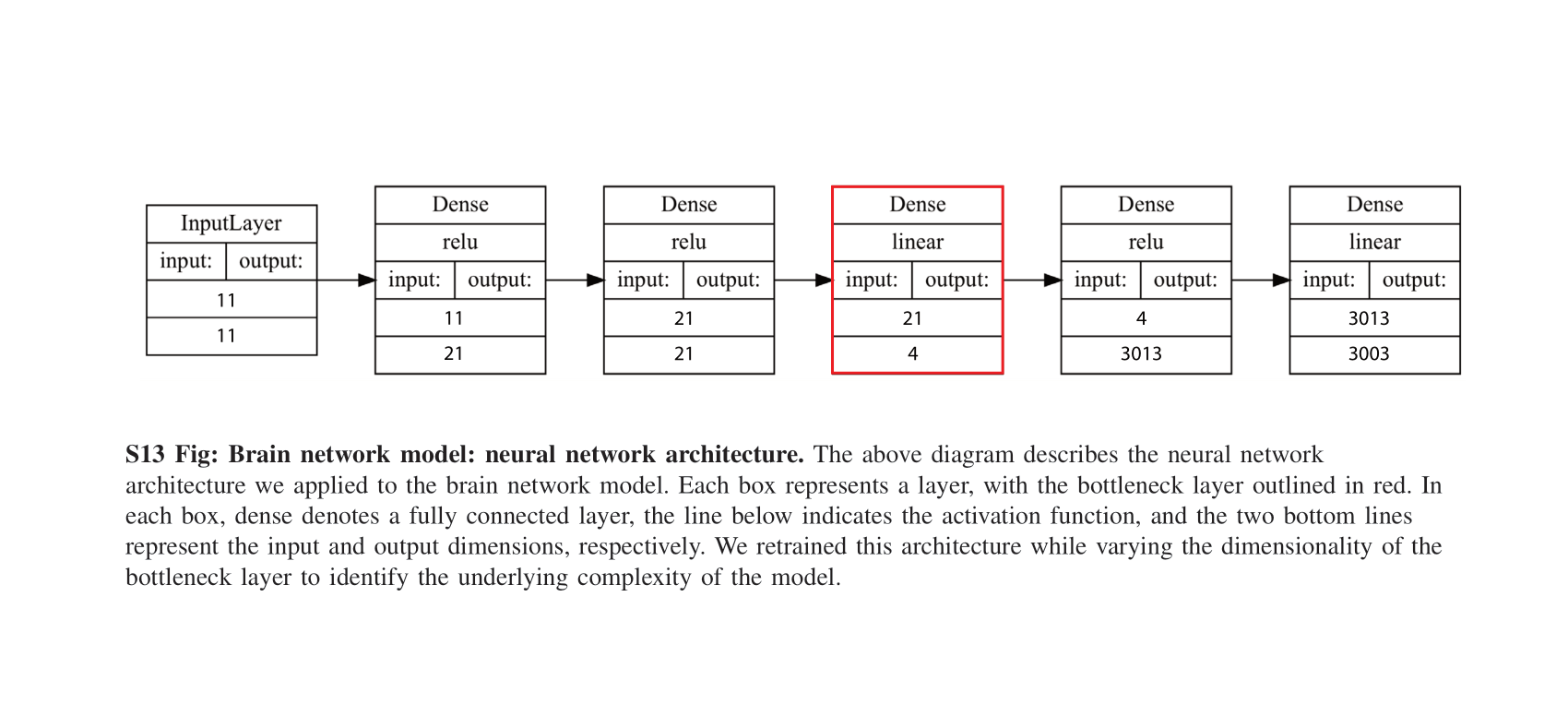

Supplement: S13 Fig — (TIFF) [file pcbi.1012283.s013.tiff]

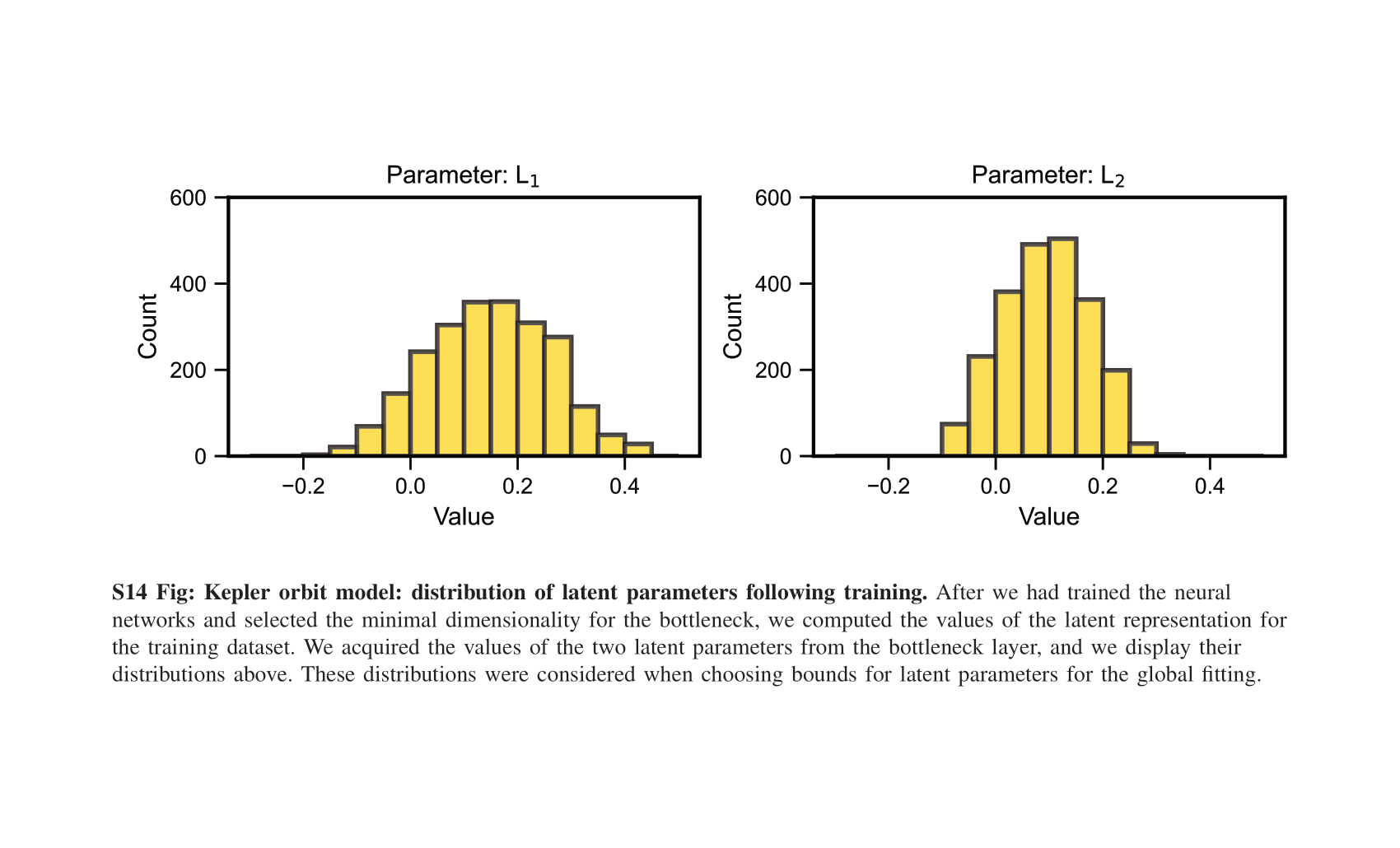

Supplement: S14 Fig — (TIFF) [file pcbi.1012283.s014.tiff]

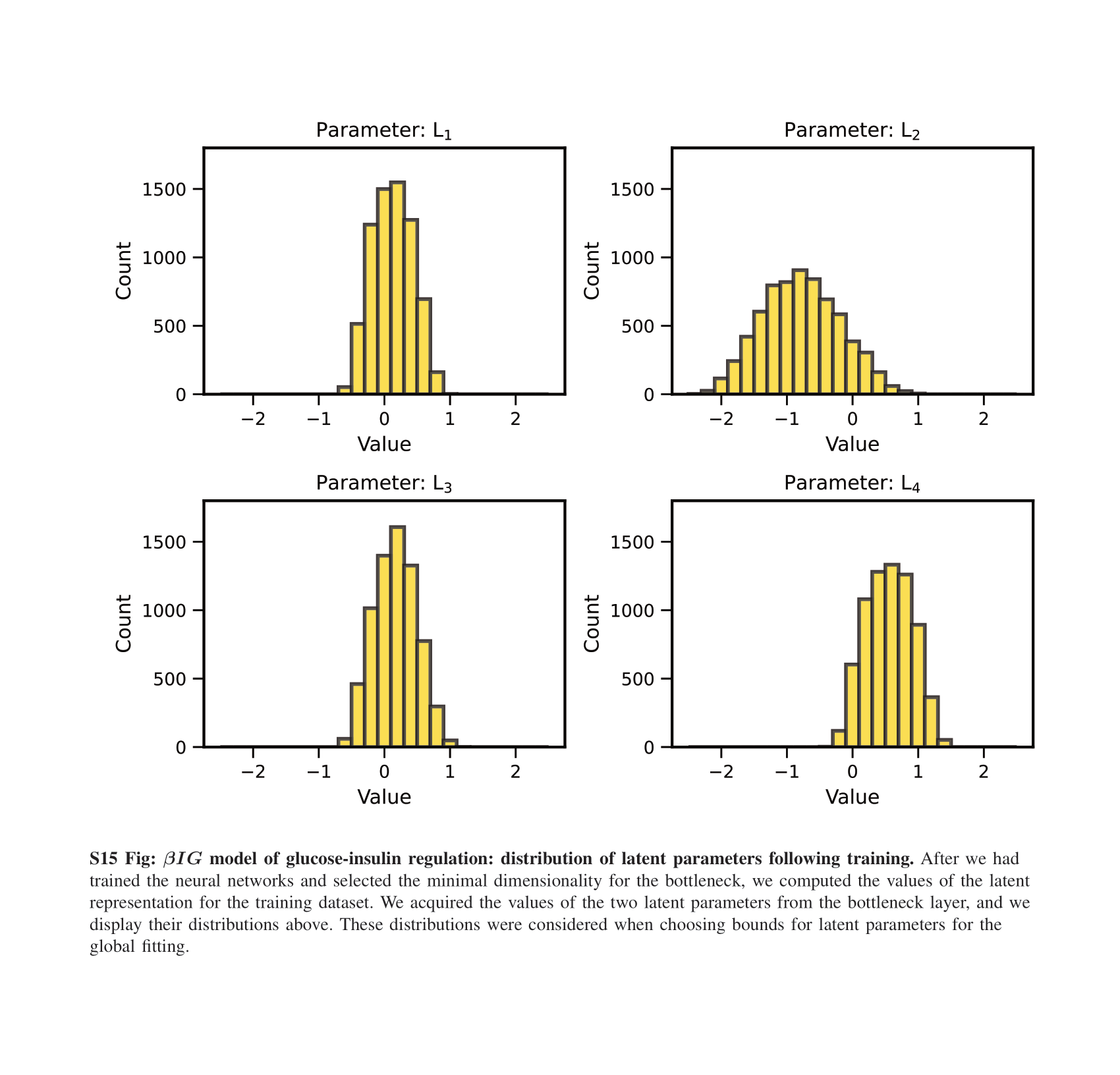

Supplement: S15 Fig — (TIFF) [file pcbi.1012283.s015.tiff]

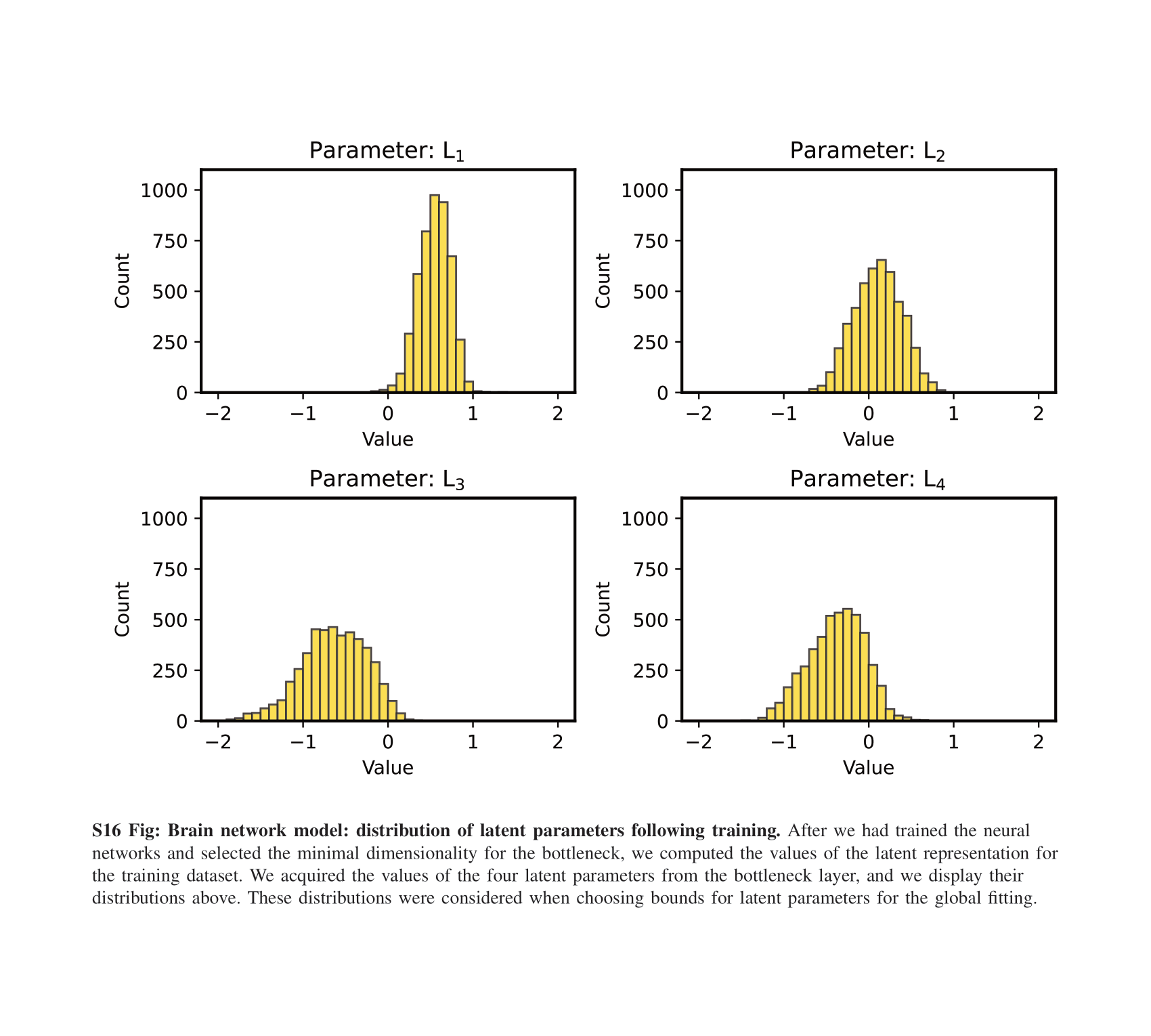

Supplement: S16 Fig — (TIFF) [file pcbi.1012283.s016.tiff]
