# Supplementary material for: Achieving Occam’s razor: Deep learning for optimal model reduction
Source: PLoS Comput Biol. 2024 Jul 18;20(7):e1012283. doi: 10.1371/journal.pcbi.1012283 (PMC11288447; doi:10.1371/journal.pcbi.1012283)
Supplement: S1 Table — (PDF) [file pcbi.1012283.s017.pdf]

S1 Table: Parameters of the Larter-Breakspear model with inclusion/exclusion criteria for this study. *has remained constant since initial implementation, see [1], [2], [3]

Parameter	Description	Included	Reason for exclusion
V_{Na}	Na^{+} reversal potential	Yes	
V_{K}	K^{+} reversal potential	Yes	
V_{Ca}	Ca^{2+} reversal potential	Yes	
V_{L}	Leak channels reversal potential		Partially phenomenological (sum of other currents) so difficult to interpret* [4]
g_{Na}	Na^{+} conductance	Yes	
g_{K}	K^{+} conductance	Yes	
g_{Ca}	Ca^{2+} conductance	Yes	
g_{L}	Leak channels conductance		Partially phenomenological (sum of other currents) so difficult to interpret* [4]
T_{Na}	Na^{+} channel threshold		No biological reason to believe that threshold is impacted (function of protein).*
T_{K}	K^{+} channel threshold		No biological reason to believe that threshold is impacted (function of protein).*
T_{Ca}	Ca^{2+} channel threshold		No biological reason to believe that threshold is impacted (function of protein).*
δ_{Na}	Na^{+} channel threshold variance		No biological reason to believe that threshold is impacted (function of protein).*
δ_{K}	K^{+} channel threshold variance		No biological reason to believe that threshold is impacted (function of protein).*
δ_{Ca}	Ca^{2+} channel threshold variance		No biological reason to believe that threshold is impacted (function of protein).*
V_T	Excitatory neuron threshold voltage		Fixed at 0.0 due to normalized voltage space [1]
Z_T	Inhibitory neuron threshold voltage		Fixed at 0.0 due to normalized voltage space [1]
δ	Variance of thresholds	Yes	
$Q_{V_{\text{max}}}$	Excitatory population maximum firing rate		No biological reason to believe that the inherent upper bound will vary.*
$Q_{Z_{\text{max}}}$	Inhibitory population maximum firing rate		No biological reason to believe that the inherent upper bound will vary.*
a_{ee}	Excitatory-to-excitatory synaptic strength	Yes	
a_{ei}	Excitatory-to-inhibitory synaptic strength	Yes	
a_{ie}	Inhibitory-to-excitatory synaptic strength		Phenomenological inhibitory mass, so no exact biological interpretation. [4]
a_{ne}	Non-specific-to-excitatory synaptic strength		Scaling factor for driving current (see I_0).*
a_{ni}	Non-specific-to-inhibitory synaptic strength		Scaling factor for driving current (see I_0).*
I_0	Subcortical excitatory input		Necessary driving current for model, but not resolvable experimentally. [1], [4]
b	Time scaling factor		Scaling factor to normalize parameters - not biologically detailed [1]
ϕ	Temperature scaling factor		Scaling factor to normalize parameters - not biologically detailed [1]
τ_{K}	K^{+} relaxation time		Time constant for potassium dynamics to convert to normalized space [1], [3]
r_{NMDA}	NMDA/AMPA receptor ratio	Yes	
c	Global region-to-region coupling constant	Yes	

References

1. Breakspear M, Terry JR, Friston KJ. Modulation of excitatory synaptic coupling facilitates synchronization and complex dynamics in a biophysical model of neuronal dynamics. Network: Computation in Neural Systems. 2003;14(4):703.
2. Endo H, Hiroe N, Yamashita O. Evaluation of resting spatio-temporal dynamics of a neural mass model using resting fMRI connectivity and EEG microstates. Frontiers in computational neuroscience. 2020;13:91.
3. Chesebro AG, Mujica-Parodi LR, Weistuch C. Ion gradient-driven bifurcations of a multi-scale neuronal model. Chaos, Solitons & Fractals. 2023;167:113120.
4. Larter R, Speelman B, Worth RM. A coupled ordinary differential equation lattice model for the simulation of epileptic seizures. Chaos: An Interdisciplinary Journal of Nonlinear Science. 1999;9(3):795–804.
