# Supplementary material for: Achieving Occam’s razor: Deep learning for optimal model reduction
Source: PLoS Comput Biol. 2024 Jul 18;20(7):e1012283. doi: 10.1371/journal.pcbi.1012283 (PMC11288447; doi:10.1371/journal.pcbi.1012283)
Supplement: Attachment — Submitted filename: response_to_reviewers.pdf [file pcbi.1012283.s018.pdf]

Response to Reviewers

Dear Editors,

We appreciate the time you and the reviewers spent patiently and thoroughly reviewing this manuscript. We have now made the requested revisions and doing so has significantly improved the paper. We have also included a new example on real data to better demonstrate the generalizability of our approach and its applicability across a wider range of scientific fields. Please find below a point-by-point response to all comments. We have included a detailed tracked-changes document to facilitate the identification of all cited changes.

Sincerely,

Corey Weistuch

Reviewer 1

Antal et al propose a novel use of deep learning for parameter identification in nonlinear dynamical systems. This is certainly an intriguing proposal and the paper plus proof-of-principle illustrations are well presented. However I feel the core algorithm needs to be more deeply exposed, broader in silico validation should be presented; and comparison with existing techniques should be performed and not just discussed. These issues are outlined in further detail below. If addressed, I think the paper could make an interesting and useful contribution to PLoS CB.

1/a The overall architecture of the inference method is well presented in Figure but not the details evaded me. I assume that forward in silico simulations of the generative model over a range of parameter values are performed, and the timeseries from each run are fed through the neural network. I assume the neural network knows nothing about the generative model but rather (using the black box mechanism of multilayer NN's) just learns the dimension == bottleneck dimension across the "landscape" of simulations. Some actual realizations of the technique in supplementary figures would be helpful.

We have introduced a new supplementary figure (Figure S1), presenting a step-by-step guide to using our tool. Its caption includes pointers to corresponding methods sections, providing a quick reference to technical details.

1/b What about the observation function (e.g. the BOLD convolution in the neuronal/functional connectivity example)?

We used a standard implementation of the Balloon-Windkessel model to transform simulated neuronal signals to BOLD signals in our work (see Methods 5.1.3 and 5.2.3). We omit the equations for the model as they are identical to prior implementations. While it is possible to use FixFit to examine the Balloon-Windkessel model (or another BOLD signal approximation) for additional redundancies, this is beyond the purpose of this example.

2/a I'm not sure that 2 noise free examples are sufficient; a jump from a simple low dimension mechanical system to a complex high dimensional system. What about forward modelling of a bilinear or even first order nonlinear model (such as a 2 node nonlinear DCM [1])?

Using a 2-node DCM example is not optimal for showcasing our method because it lacks any anticipated parameter redundancies. Instead, we have applied FixFit to an additional biological example of glucose-insulin-beta-cell regulation. This provides an intermediate model with sufficiently complex dynamics (dynamical compensation, external varied inputs, non-linear

feedback). In this example, we fit the representation earlier identified by FixFit to experimental data.

Karin, Omer, et al. "Dynamical compensation in physiological circuits." Molecular systems biology 12.11 (2016): 886.

2/b And what is the role of observation noise, especially if auto-correlated – these are the sorts of challenging counterexamples of classic nonlinear time series methods stumbled through very poorly [2].

We have used the new intermediate complexity example to demonstrate that FixFit can be used as part of a pipeline to fit real data with observational noise. We have also added a new discussion paragraph to elaborate on the roles of both observational and model noise.

3. The conceptual comparison with Bayesian methods on p11 was very interesting but I think a 1 to 1 actual comparison is required. For example, using variational Bayes with Free Energy for model comparison can deal with some of the problems identified here, including conditional dependences between system parameters. By including an observation model with measurement noise priors, these approaches have been shown to work in the sort of deeper in silico validations I think are required here (see [1,3-5] for example).

We have now expanded our discussion of how FixFit and Bayesian methods complement rather than compete. FixFit does not directly fit the data itself, instead, it reparameterizes the model by eliminating redundancies. Subsequently, the model can be fitted to data using Bayesian inference. The reparameterization is often necessary because conventional Bayesian sampling methods, like MCMC, cannot appropriately sample flat posteriors, such as those resulting from structural redundancies. Similarly, variational Bayes with Free Energy requires an approximately Gaussian posterior distribution (finite variance), and since perfect redundancies will have infinite variance, this core assumption is violated. FixFit is designed to overcome this and identify a simplified, redundancy-free model. The new reduced model can then be fitted using standard parameter fitting strategies. While we used a global optimizer in our examples, any parameter fitting strategy, including a Bayesian approach, can naturally replace it.

Reviewer 2

In their paper "Achieving Occam's razor: Deep learning for optimal model reduction" , Antal and colleagues present "FixFit" a method that uses simulation of dynamical systems together with bottleneck DNN (similar to autoencoders but mapping from parameters to data/timecourses instead of data to data). By finding the smallest bottleneck dimension that gives a validation

accuracy close to the best, they can determine the dimensionality of the system simulations. The bottleneck layer of the DNN automatically defines a set of latent parameters. Once the network is learned, it can be used to fit new data and the authors can inspect the sensitive of latent parameters with respect to the original parameters of the system. The authors demonstrate the dimensionality reduction on to systems, a "simple" Kepler orbit equation and a higher dimensional dynamical system of neural activity.

This paper introduces an interesting method that could be very useful for many subfields of neuroscience, where models are often complicated, and it is not trivial to understand dependencies among parameters. Here, FixFit provides a method to analyse the model and its output and find the "minimal" amount of parameters needed to span the model output. However, the dimensionality reduction is based on simulated data and thus does not necessarily reflect the dimensionality of acquired data. In other words, Occam's razor, does not depend on the data. In that sense, an application to real data is missing.

The paper is well written, although I think that the methods, in particular, the description of global fitting, would profit from more detail.

Overall, my assessment is very positive, but I think that the paper would profit from an illustration on real data and from some simulation that underlines the claims about noise. See my comments below.

Major:

1. Occam's razor for real data?: While you illustrate how you can use FixFit to recover the minimal dimensionality needed to (re)cover simulations drawn from the entire parameter space (with some restrictions, see below), it is not clear to me how you would apply Occam's razor on real data. You could create a Bottleneck network and then fit to real data. It would be really good, if you could show this. It should not be difficult to get these data, e.g. for the resting state FC. However, how would you determine then, whether the model has the right dimensionality for the real data? Would you need to retrain your bottleneck network on real data? One could also think of this as the following question: Is there a way of assuring that your latent space dimensionality is the dimensionality of what your model can explain in the real data? Could that dimensionality be even lower? Even if you are not able to solve this, it would be good to discuss it and to show an application to real data.

Our work now includes a third example where we show that the latent parameter representation learned by FixFit can be fit to real data. The bottleneck network determines the number of independent control knobs a given model has, independent of real data. FixFit thus determines the maximum number of parameters that can be extracted from measured data, given that the

underlying model is correct. When the reduced model produced by FixFit is fit to noisy data using traditional parameter estimation strategies (e.g., Bayesian, least-square, etc), the set of extractable parameters can be smaller.

2. Data fitting: It was not clear to me how the "data" fitting procedure was performed. I understand that this is done at then end, once a bottleneck network is trained and validated. From this network you then try to infer the latent states and parameters. Which data were used for the global fitting analysis? New simulations, or one of the data points used for training or validation of the DNN. Are the points displayed in figures 2C and 2D different data points or different solutions when fitting one single simulated orbit? I assume it is one orbit. Why did the optimizer evaluation stop at solutions that are not fitting well? Or what do you mean by optimizer evaluation? As said, I would assume that global fitting happens on a learned network. You say, that the algorithm you apply does not guarantee to find the global optimum. Could you elaborate on this? Does this imply that it is still very difficult to find the best and, therefore, the unique solution, which you want to find (according to the abstract). One of the reasons for reducing the parameter dimensionality is to avoid problems for fitting when the landscape can show flat ridges etc due to correlated parameters. Please comment on this.

The samples utilized for global fitting analysis varied between our two original examples. This is now stated in the results section for each example. For the Kepler orbit example, we employed simulated samples from the same set used during the DNN training. This differed in the case of the brain network model. Here, we fitted two samples differing only in a single parameter value. As such pairs did not exist on the original Sobol sampling grid, we newly simulated these two samples for the fitting process. Consequently, they were not part of the DNN training dataset. We have introduced a third example (BIG model) where we fitted real data. Collectively, these examples showcase that our method's representation can be effectively fitted to simulated training/test and real data.

The points shown in figure panels 2C and 2D are individual evaluations of the objective function which were performed by the optimizer throughout an optimization run. We now specify that the dark blue points correspond to the global optima identified by the optimizer. Both panels show results derived from fitting the same orbit. The algorithm had no difficulty in finding the global optimum in our example cases.

More broadly, the global optimizer that we employ in our analyses uses a basin-hopping algorithm to find a global optimum among local minima. If the objective landscape is very bumpy, or the global minimum has a very narrow local environment, the optimizer may still fail to find the global optimum. This can be prevalent in the case of redundant parameters where the irregular landscape will trap the optimizer in local optima before it can reach one of the many equally good global

optima. Latent parameter space, due to the lack of ridges and other features of redundancy, will always be easier for optimizers to explore.

3. Noise: In the introduction you write about how FixFit could be used "to gauge how resolvable complexity depends on the amount of noise." I think this is very interesting, but I was missing any consideration of noise in your results/simulations. It was not clear to me, how you would actually do this. I think it would be good to illustrate this gauge effect in a simulation/application. Is the bottleneck embedding sensitive to noise, or not? How can applying FixFit only on simulated data (without noise) help with this gauge?

We have added a new paragraph to the discussion section that elaborates on the impact of both observational and model noise. In brief, observational noise broadens the confidence intervals for model parameters but has no effect on system dynamics. Thus, the structural redundancies identified by FixFit remain unchanged. Instead, traditional techniques (confidence intervals, Bayesian statistics) can be used on the reduced model generated by FixFit to determine the information content of real data (i.e. the parameter variance).

For systems with model noise (such as Langevin dynamics), a recurrent neural network with a bottleneck layer could be trained to generate stochastic trajectories dependent on model parameters. These stochastic effects do alter system dynamics, which may affect the number of latent parameters identified by FixFit. However, we believe that investigating these effects goes beyond the scope of our current work and warrants a more in-depth follow-up study.

Minor:
4. You present two very specific bottleneck-network architectures, presumably optimized for the two example cases (dynamical systems). How to choose the correct/best network with bottleneck? Does it matter which bottleneck network one choses? I think it would be good to at least briefly discuss this.

We have now added an additional discussion paragraph to provide guidance on how to choose the best neural network architecture.

5. Heuristic to determine bottleneck size: You define the bottleneck dimension as the lowest dimension with a fit not significantly worse than the best. In the two cases you present, this dimension is very clear and also observable as a knee. Would you expect this always to be the case (also for other models) and to what degree could noise flatten the curve and thus make it more difficult to define the bottleneck dimension. Could you give the reader some hint/indication towards this problem?

The chosen heuristic makes use of the model validation error, a common metric for determining the degree to which a model is overfit to training data. Thus, the widespread use of this metric suggests that our heuristic will generalize well to other examples. As for how noise may influence the model's dimensionality and more broadly parameter identifiability, we explicitly elaborate on these aspects in the new discussion paragraph focusing on the role of noise.

6. Fixing parameters in the multi-scale model (line 272ff): You fix 19 parameters that you "determine to be unlikely to be sensitive". Who did you determine those? Wouldn't it be exactly the purpose of FixFit to actually determine these parameters?

In principle, we agree that FixFit will be able to detect the relative sensitivity of the model to these parameters. However, many parameters in the LB model are either unlikely to change across real physiological conditions or lack a clear biological interpretation. To manage the computational costs of this example, we focused on the parameters that we believed were most relevant to the community. We have added additional clarification on a parameter-by-parameter basis in Supplemental Table 1.

7. Line 66/67: How is Figure S2 related to global optimization and unique solutions? It shows the distribution of latent parameters. Please clarify.

We used these distributions to establish bounds for the parameter values during global optimizations. To clarify their purpose, we have moved the references to SI figures S14 and S16 (originally S2 and S4) to the Methods, under Global Fitting.

8. Supplementary Figures S10 and S11: Please provide more information in the caption for readers who are not familiar with the notation you have chosen. I understand that the two lowest lines are input and output dimensions. Is this correct. It would also be nice, if you could highlight the bottleneck layer.

We thank the reviewer for pointing out this oversight. The captions of these figures now contain complete descriptions of each element of these figures. We have now also highlighted the bottleneck layer.

9. Please specify what a ReLu activation is.

This is now explained in the methods section 5.3.2.
